# Attainment of Quiet Standing in Humans: Are the Lower Limb Joints Controlled Relative to a Misaligned Postural Reference?

**DOI:** 10.3389/fphys.2019.00625

**Published:** 2019-06-06

**Authors:** Irene Di Giulio, Vasilios Baltzopoulos

**Affiliations:** ^1^School of Basic and Medical Biosciences, Faculty of Life Science and Medicine, King’s College London, London, United Kingdom; ^2^Research Institute for Sport and Exercise Sciences, Liverpool John Moores University, Liverpool, United Kingdom

**Keywords:** human stance control, standing balance, neural control of movement, lower limb joints, body misalignment to line of gravity, initiation of standing

## Abstract

In human quiet standing, the relative position between ankle joint centre and line of gravity is neurally regulated within tight limits. The regulation of the knee and hip configuration is unclear and thought to be controlled passively. However, perturbed standing experiments have shown a lower limb multi-joint coordination. Here, measuring the relative alignment between lower limb joints and the line of gravity in quiet standing after walking, we investigated whether the configuration is maintained over time through passive mechanisms or active control. Thirteen healthy adults walked without following a path and then stood quietly for 7.6 s on a force platform (up to four trials). The transition between initiation and steady-state standing (7.6 s) was measured using motion capture. Sagittal lower limb joint centres’ position relative to line of gravity (CoG_AP_) and their time constants were calculated in each trial. Ankle, knee, and hip joint moments were also calculated through inverse dynamics. After walking, the body decelerated (τ = 0.16 s). The ankle and hip joints’ position relative to CoG_AP_ measured at two time intervals of quiet standing (Mid = 0.5–0.55 s; End = 7.55–7.6 s) were different (mean ± SEM, CoG_AP–Ankle_Mid_ = 47 ± 4 mm, CoG_AP–Ankle_End_ = 58 ± 5 mm; CoG_AP–Hip_Mid_ = 2 ± 5 mm, CoG_AP–Hip_End_ = −5 ± 5 mm). The ankle, knee, and hip flexion-extension moments significantly changed. Changes in joints position relative to CoG_AP_ and misalignment suggest that joint position is not maintained over 7.6 s, but regulated relative to a standing reference. Higher joint moments at steady-state standing suggest mechanisms other than passive knee and hip regulation are involved in standing.

## Introduction

Human standing is an everyday activity, and it constitutes the mechanical and control basis for other movements, such as gait and reaching. In quiet standing, the body is unstable in the sagittal plane (Morasso and Schieppati, 1999; Loram et al., 2007; Kiemel et al., 2011). To maintain standing successfully, the location of the body centre of mass and of the ankle joint relative to the line of gravity need to be regulated via neural feedback control (Peterka and Benolken, 1995; Loram and Lakie, 2002; Loram et al., 2007).

Because the body centre of mass sway range is only 18–21 mm in standing (Gatev et al., 1999), whole body configuration (i.e., the position of body segments and joints relative to the line of gravity) must to be tightly regulated. The traditional understanding is that in quiet standing, only the ankle position needs to be regulated. The knee and hip positions relative to the line of gravity are thought to be either passively determined taking advantage of the close packed position (Steindler, 1964, pp. 330–349; MacConaill and Basmajian, 1977, pp. 31–52), or tonically but not phasically regulated (Steindler, 1964, pp. 106–108, 110–114). Essentially, it was accepted that there is no need for a modulation of knee and hip moments in quiet standing.

More recent work has shown that ankle, knee, hip, L5-S1 joint (fifth lumbar and first sacral vertebrae), C7-T1 joint (seventh cervical and first thoracic vertebrae) and atlanto-occipital joint are controlled in a coordinated fashion in standing (Hsu et al., 2007) according to the uncontrolled manifold analysis. Focusing on the hip joint, Kiemel et al. (2008) showed that intrinsic stiffness is not enough for hip passive stability and neural control is required to maintain standing. Furthermore, ankle, knee, and hip joints showed a multi-joint coordinated behavior in perturbed standing (Di Giulio et al., 2013). When gentle knee perturbations were applied at the knee, if the knee displacement after perturbation was small, also ankle and hip displacements were reduced and the whole lower limb was stiffer (locked or inverted-pendulum like). On the other hand, when the knee displacement after perturbation was larger, ankle and hip displacements were also larger and the whole lower limb did not show an inverted pendulum-like configuration. This suggests that lower limb joints’ stiffness or mobilization is controlled collectively, and even the knee joint is not necessarily passively locked. What remains an open questions is whether this inter-joint relationship is purely mechanical or tonic or whether phasic control is involved.

We designed an experiment that substantially changed body configuration in order to measure how joint position in relation to line of gravity was attained in the transition to quiet standing. Gait before quiet standing was used to measure standing initiation and configuration changes to achieve quasi-static equilibrium. We did not use non-ecological perturbations, such as platform translations or tilts, to avoid the introduction of artificial responses and habituation to the perturbation over time. Our approach was to study the transition of joint position between initiation and steady-state standing and analyze which factors could explain the process. By studying initiation of standing and transition to steady-state standing, we asked (i) What is the relative alignment between lower limb joints and the line of gravity? (ii) Is the lower limb configuration at steady state standing determined by the position at initiation of standing or is it actively controlled and corrected?

One could expect that biomechanical (e.g., body deceleration to stop the body after walking) and passive mechanisms (e.g., stiffness) could wholly explain the joint position at initiation and steady-state standing. An additional expectation is that steady-state standing configuration could depend on the body configuration at initiation of standing and no corrections are occurring as long as standing is successful and efficient. Furthermore, if optimization and energy cost minimization was a principle of standing regulation, steady-state standing configuration should be consistent with reduced muscular effort. This would suggest that steady-state standing configuration is aligned with the vertical to reduce load on the joints and the need for phasic muscular activation to maintain balance.

On the other hand, if lower limb configuration changed during standing, we could investigate whether the difference in lower limb configuration between initiation and steady-state standing was consistent with energy cost minimization (i.e., the joints became more aligned) or not. We could also measure whether the steady-state lower limb configuration was dependant on the initial variable condition established by gait (i.e., not repeatable and inconsistent across trials).

In this study, we measured the lower limb joint alignment with line of gravity in quiet standing and we investigated the mechanisms involved in this task. Understanding whether alignment was maintained or corrected would indicate whether passive stiffness or other mechanisms to control the lower limb joints are involved in standing.

## Materials and Methods

### Ethical Approval

Participants gave written informed consent to these experiments which were approved by the ethics committee of the Institute for Biomedical Research into Human Movement and Health, Manchester Metropolitan University and conformed to the standards of the Declaration of Helsinki.

### Participants and Procedures

Thirteen participants (age 46 ± 13 years, mass 71.7 ± 13.0 kg, height 1.68 ± 0.13 m, seven women and six men) who self-reported no neurological or musculoskeletal injuries or disorders took part in this study.

The data reported here is part of a larger experiment that lasted about 3 h. For each participant, the session was structured as follows. Participants arrived to the laboratory and informed consent was obtained (10 min). Bilateral knee and hip MRIs were collected (60 min) and markers were placed on participant’s anatomical landmarks (30 min). The first two trials of the current experiment (5 min) were collected and then an intervening knee perturbation experiment (40 min including EMG placement, as reported in Di Giulio et al., 2013) was conducted. The remaining trials of the current experiment were recorded after that (5 min), and a final experiment on control of standing with another set-up (30 min) was recorded. Breaks were also allowed between trials and experiments.

For this experiment, participants walked randomly for a few seconds around the laboratory without following a particular path and ended their walk anywhere on a force plate (508 mm × 464 mm) with feet broadly symmetrical (about shoulder width, feet broadly parallel to each other). Some participants walked over an imaginary circle or ellipsoid, others walked on a straight line, others turned around and changed direction at least once. Participants were asked to approach the force platform in a straight line (last 1–2 steps). The operator monitored the participant’s gait phase in order to start the recording timely, and the trial was repeated if the participants did not approach the platform complying with this criterion. Participants were not asked to replicate their walking path and most of them completely changed it over different trials. Participants were asked to end their walking phase in a comfortable and usual manner, and stand normally looking in front of them.

**FIGURE 1:**
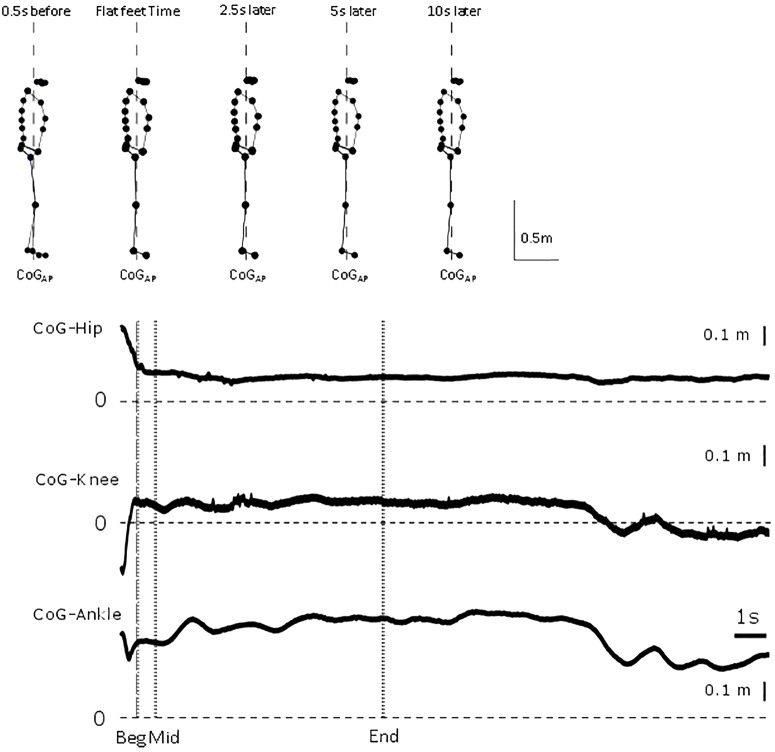
Representative trial. From top to bottom, whole body sagittal stick figure from markers location of representative participant at 0.5 s before flat feet time, flat feet time, and 2.5, 5 and 10 s after flat feet time. Vertical dashed line represents line of gravity location. For the representative trial, relative displacement between CoG and hip, knee and ankle position between 0.5 s before flat feet time and end of trial. Vertical dashed line represents flat feet time, dotted lines identify the intervals for which differences were calculated in the analysis.

The recording was manually initiated when the participant approached the force plate and each trial lasted 30 s (from when the participant approached the force plate). Because of this variable trial start, different effective standing durations were recorded. For analysis, the longest common duration of standing after its initiation (flat feet time, see below) was used (7.6 s, see Figure 1). Although a longer common duration was not possible, 7.6 s after standing initiation is likely to be sufficient to measure changes due to and possibly beyond body deceleration, without fatiguing the participants. All the participants performed at least four trials (with intervening break). When technical problems were identified in real time, the trial was repeated. However, other technical difficulties in the markers trajectories reconstruction were only identified during data processing and those trials were not included in further analysis. For this reason, out of the thirteen participants, we could use 4 trials for seven participants, 3 trials for three participants and 2 trials for three participants. These participants were included in the analysis because more than one repetition was available, to include as much population variability as possible, and because the data included was highly reliable and accurate thanks to the precision of the techniques (motion capture and force plates) and the corrections adopted from the MRI scans (marker positioning and correction of joint centres calculation).

### Apparatus and Measurements

#### Imaging

Four MRI scans were collected with the participants in the standing position (G-Scan, Esaote, Genoa, Italy) to improve joint location accuracy. The same protocol was used for knee and hip joints bilaterally: spin T1-weighted HF, matrix 256 × 256, coronal and transverse planes. Slice thickness and the inter-slice gap were 0.4 and 4 mm for the knees, and 0.6 and 6 mm for the hips. Cod liver oil pills were placed on anatomical landmarks where the retro-reflective markers would be placed for motion analysis. If the image showed that the cod liver oil pill was not placed correctly, it was replaced accordingly and another set of scans was collected. This accurate location was then used to place the motion analysis marker.

#### Motion Capture

A 10 camera motion analysis system (VICON 612, Oxford Metrics, United Kingdom) was used to measure body kinematics. Retro-reflective markers were placed on the sacrum, third lumbar vertebral process (L3), twelfth, tenth, seventh, and third thoracic vertebral process (T12, T10, T7, T3), seventh cervical vertebral process (C7), and sternum and clavicle. Other markers were placed bilaterally on the first, second, and fifth metatarsal head, the lateral and medial malleolus, the heel, the tibia (for 3D segment definition), and the most prominent points of the lateral and medial tibial condyles, the lateral and medial femoral epicondyle, the greater trochanters, the anterior and posterior iliac spines, the zygomatic process anterior to the auditory meatus, and the temporal process of the zygomatic bone (at the inferior margin of the ocular orbit). After walking, participants stood with both feet on a force plate (AMTI, OR6-7, Watertown, MA, United States). The Ground Reaction Force (GRF) and its point of application were recorded. Kinematic and force plate data were sampled at 60 Hz.

### Data Analysis

The following analysis was performed using MATLAB (MathWorks, Natick, MA, United States).

**FIGURE 2:**
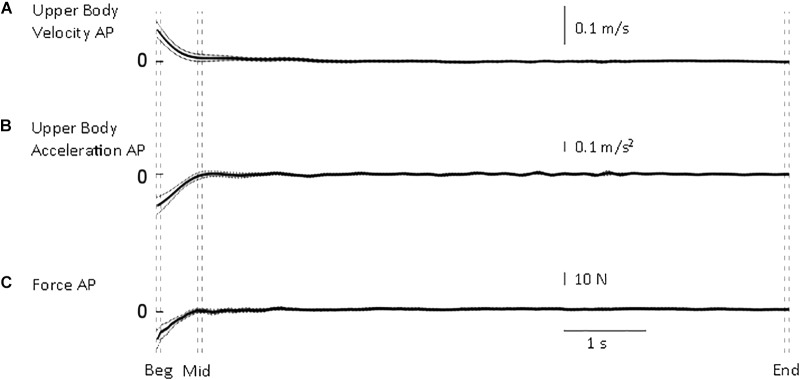
Whole body quantities. From top to bottom, mean (solid) ±95% confidence intervals (dotted) of **(A)** upper body antero-posterior velocity **(B)**, deceleration **(C)** and shear force from the force platform. Body velocity was quickly reduced to approximate the end of standing velocity. Vertical dashed lines illustrate the three intervals used in the statistical analysis: beginning of standing (“Beg” from flat feet time *t* = 0–0.05 s later), after body deceleration has ceased (“Mid” 0.5–0.55 s after flat feet time) and steady-state standing (“End”, 7.55–7.6 s after flat feet time).

**FIGURE 3:**
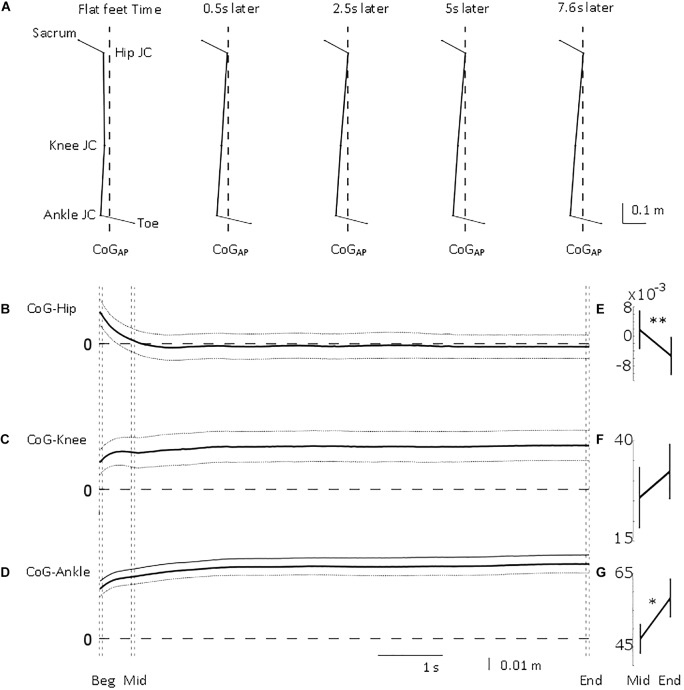
Antero-posterior lower limb joint position relative to centre of gravity position. From top to bottom, **(A)** mean stick figure obtained from sagittal position of lower body markers’ locations (toe and sacrum) and calculated joint centres (ankle, knee, and hip) relative to line of gravity location (dashed) from all the trials at five time points (flat feet time, 0.5, 2.5, 5, and 7.6 s after flat feet time). Mean (solid) ±95% confidence intervals (dotted) of **(B)** hip, **(C)** knee, and **(D)** ankle joint centre location relative to centre of gravity position for the common duration to all the trials included in the analysis (i.e., 7.6 s). 0 m represents perfect sagittal alignment between joint centre and gravity. Vertical dashed lines illustrate the three intervals used in the statistical analysis: beginning of standing (“Beg” from flat feet time *t* = 0–0.05 s later), after body deceleration has ceased (“Mid” 0.5–0.55 s after flat feet time) and steady-state standing (“End”, 7.55–7.6 s after flat feet time). Mean ± standard error of the mean at Mid and End intervals for **(E)** CoG-Hip, **(F)** CoG-Knee, and **(G)** CoG-Ankle. ^∗^*p* < 0.05; ^∗∗^*p* < 0.01.

**FIGURE 4:**
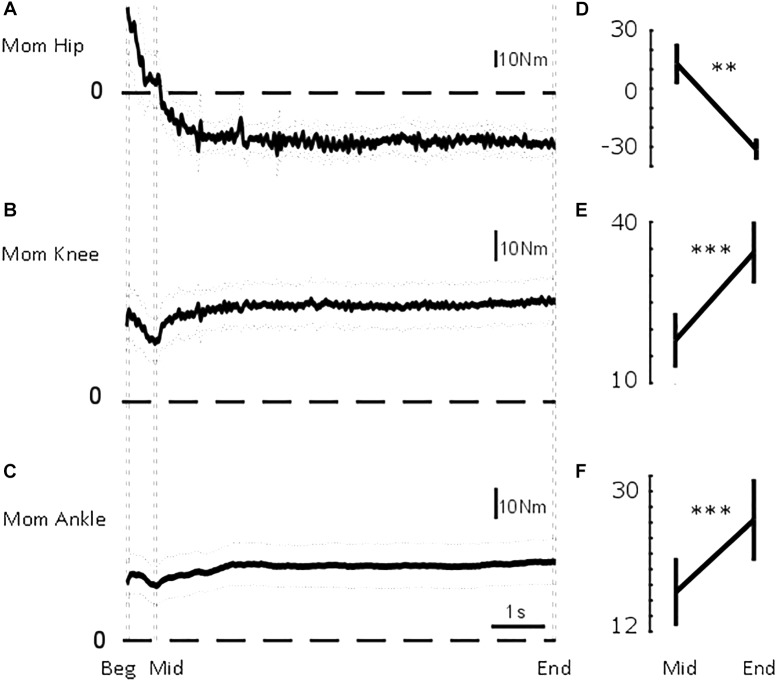
Sagittal lower limb joint moments. From top to bottom, mean (solid) ±95% confidence intervals (dotted) of **(A)** hip, **(B)** knee, and **(C)** ankle sagittal moments for the common duration to all the trials included in the analysis (i.e., 7.6 s). Vertical dashed lines illustrate the three intervals used in the statistical analysis: beginning of standing (“Beg” from flat feet time *t* = 0–0.05 s later), after body deceleration has ceased (“Mid” 0.5–0.55 s after flat feet time) and steady-state standing (“End”, 7.55–7.6 s after flat feet time). Mean ± standard error of the mean at Mid and End interval for **(D)** hip flexion-extension moment, **(E)** knee flexion-extension moment, and **(F)** ankle dorsi-plantarflexion moment. ^∗∗^*p* < 0.01; ^∗∗∗^*p* < 0.001.

For each trial, the last heel-ground contacts during walking and prior to standing were calculated for left and right foot as the instant of minimum velocity of the toe marker (Pijnappels et al., 2001), and then classified as last heel and penultimate heel contact, irrespective of the side. After the last heel contact, for each trial, the instant of last toe down was identified using the time when the velocity of the toe marker first crossed the zero value. This instant was deemed to be the start of standing, since both feet were on the ground and no further steps were taken (Figure 1). This instant is called flat feet time, 0 s in all mean data figures (Figure 2–4). To accept a trial for analysis, a test was used to confirm that flat feet time represented standing: the vertical component of the GRF had to be within one SD (±1.7 N) of the value during sustained standing (7.6 s later).

The following variables were calculated.

*Upper body velocity and acceleration*. The location of the markers from the pelvis upward was averaged to provide a representative antero-posterior body location, which was differentiated to provide velocity and acceleration, using a FIR filter (Remez differentiator pass-band filter with pass frequency 1 Hz, and stop frequency 6 Hz). The whole trial (30 s) was then reduced to the correct 7.6 s from flat feet time, removing the appropriate initial and final part of the recording and avoiding any filtering distortion at the beginning or end of the trial.

*Antero-posterior centre of gravity (CoG_AP_) location*. Antero-posterior centre of gravity (CoG_AP_) was calculated by zero-lag low-pass filtering the sagittal component of the centre of pressure (from point of application) with a cut-off frequency of 0.5 Hz (Caron et al., 1997; Loram and Lakie, 2002). This calculation is valid for and presented only during standing. We used this quantity to minimize the possible bias induced by modelling different body shapes and sizes using kinematic data.

*Antero-posterior CoG position relative to the lower limb joint centres (CoG-Ankle, CoG-Knee, and CoG-Hip)*. The displacement between a vertical line through the CoG_AP_ and the joint centres was calculated. The joint centre positions were calculated using a combination of surface markers on bony landmarks (Vicon) and MRI imaging (see section in “Apparatus and Measurements”). The ankle joint centre was calculated relative to the lateral malleolus using the individually measured ankle width. The knee joint centre was calculated as the centre of a line joining markers on lateral and medial femoral epicondyles. The hip joint centre was calculated according to the GaitLab algorithm (Vaughan et al., 1999) using three markers (sacrum, left and right anterior superior iliac spines) and anthropometric measures taken from each subject. For 10 participants (three were excluded for contraindication to MRI) the GaitLab calculation was corrected by analysis of the MRI images. The geometrical hip joint centre in the anterior–posterior direction relative to the cod liver oil pills (placed on greater trochanter and iliac spines landmarks) was calculated, approximating the head of the femur as a circular section and assuming its centre as the joint centre (Osirix 2.7.5, OsiriX Foundation, Geneva, Switzerland). The joint location used in the kinematic analysis was corrected using each participant’s difference between joint calculated from the marker and from the MRI scan. In the sagittal plane, the mean anterior/posterior correction was ±2 ± 1 mm (mean ± SD). For the participants which were excluded from MRI scans, the joint locations were not corrected and the ones calculated using anthropometry and kinematic model were used.

Left and right sagittal joint location were averaged. A displacement of 0 mm indicates that the CoG_AP_ is in line with the joint centre.

For each variable listed above and for the antero-posterior force from the force platform, a time constant was calculated for each trial between flat feet time and 7.6 s. The time constant represents the elapsed time for the system response to decay/grow by 1/*e* at the initial rate. An exponential curve was fitted to the data and the time constant was estimated for each trial and then averaged across participants.

*Joint moments (MAnkle, MKnee, and MHip)*. The flexion-extension joint moments were calculated using an inverse dynamic approach (Vaughan et al., 1999). At the ankle, positive values indicate dorsi-flexion moment, while negative values indicate plantar-flexion moment. At the knee and hip, positive values indicate flexion moments.

For each variable, the mean over three time intervals was calculated. The intervals were chosen to represent the possible phases of standing after walking. The interval duration was determined by a suitable duration that could capture the rapid changes occurring after standing initiation. Therefore, beginning of standing (“Beg”) was between flat feet time and 0.05 s later. This arbitrary choice determined the interval duration, which was kept constant. Steady-state standing (“End”) was identified as the latest interval available from the recordings (7.6 s), so that interval was between 7.55 and 7.6 s. An intermediate interval (“Mid”) was selected to start later than the threshold body acceleration time constant (0.25 s), but still adequate to record any early changes in configuration. The chosen Mid interval was between 0.5 and 0.55 s.

### Statistical Analysis

A repeated measures univariate ANOVA was run on the CoG_AP–Joints_ (generic term to indicated the displacement between CoG and the lower limb joints included in this study). Interval (3 levels), and Trial (4 levels) were fixed factors and Participant (13 levels) was the random factor. This analysis was conducted using SPSS (ver.24, IBM).

Each CoG_AP–Joint_ and joint moments were tested to see if a difference was significant between the two intervals after the deceleration had ceased (Mid vs. End) using a two-tailed paired *t*-test. We used Mid rather than Beg interval in this analysis to measure changes in configuration beyond body deceleration after walking.

Significance is reported at *p* < 0.05. Unless otherwise stated, results are reported as mean ± standard error of the mean in the text, and 95% confidence curves are shown in the figures (dotted).

## Results

All participants ended their gait with both feet flat on the force plate with a broadly symmetrical, self-chosen stance and foot placement. In Figure 1, a representative participant illustrates the small changes in configuration in a trial and show the need for high precision measurements.

The transition from walking to standing requires reduction of forward velocity and attainment of equilibrium. Following flat feet time, upper body deceleration and antero-posterior shear force are reduced to the steady-state value rapidly (Figure 2). From all trials, the time constant of the upper body deceleration was 0.16 ± 0.03 s, and a similar time constant was calculated for the shear force 0.15 ± 0.03 s (mean ± SD). These results designated τ = 0.16 s as the higher value after which body deceleration had reached a value closer to steady state.

If the main process governing the joints adjustments is only related to body deceleration, one would expect that the time constant of all the other variables to be close to τ = 0.16 s. This is a justified approach considering that body sway in quiet standing determines a not-null mean acceleration (i.e., between –0.031 and +0.035 m/s^2^ range measured in the current experiment at steady-state standing). Thus, τ = 0.16 s is consistent with the time needed for the body to approximate quiet standing. In order to define a conservative time threshold beyond which the changes measured were not merely related to body deceleration, we used the mean upper body deceleration time constant (0.16) + 3 × SD (3 × 0.03), and obtained a value of 0.25 s. Using three times SD gives our analysis 99.7% probability to be investigating adjustments that were not merely linked to body deceleration. If we found higher time constants, we were entitled to investigate the process occurring after 0.25 s.

### Is Body Configuration Only Governed by Body Deceleration?

Initially, CoG_AP_ was in front of ankle, knee, and hip by 38, 28, and 25 mm, respectively (Figure 3). The hip quickly, τ_Hip_ = 0.25 ± 0.12 s (mean ± SD), aligned more with CoG_AP_ by 27 mm (Figure 3B). The displacement between ankle and knee joint centres and CoG_AP_ increased (i.e., misalignment) by 19 and 13 mm progressively with τ_Ankle_ = 0.62 ± 0.17 s and τ_Knee_ = 0.61 ± 0.29 s (mean ± SD; Figure 3D,C). These longer time constants suggest a slower process, not related only to the deceleration of the body. The joint moments (Figure 4) showed a similar transition. The ankle and knee moments increased by 6.79 and 7.97 Nm, respectively, while the hip moment decreased by 9.78 Nm and transitioned from extension at the beginning of standing to flexion at steady-state standing.

We investigated the repeatability and trend in CoG_AP–Joints_ and moments.

*Univariate analysis*. CoG_AP–Ankle_ showed a significant difference between participants [*F*(12,21.857) = 2.722, *p* = 0.020], but no difference with trial [*F*(3,27) = 1.516, *p* = 0.233] or interval [*F*(2,24.619) = 1.255, *p* = 0.303]. An interaction interval × participant [*F*(24,54) = 6.962, *p* < 0.001] was found.

CoG_AP–Knee_ and CoG_AP–Hip_ showed a significant difference between intervals [*F*_Knee_(2,27.531) = 23.707, *p*_Knee_ < 0.001; *F*_Hip_ (2,33.559) = 52,490, *p*_Hip_ < 0.001]. CoG_AP–Knee_ and CoG_AP–Hip_ showed no difference between trials [*F*_Knee_(3,27) = 1.557, *p*_Knee_ = 0.223; *F*_Hip_(3,27) = 0.045, *p*_Hip_ = 0.987] or participants [*F*_Knee_ (12,14.007) = 1.437, *p*_Knee_ = 0.256; *F*_Hip_(12,5.713) = 0.602, *p*_Hip_ = 0.785].

Ankle, knee, and hip moment (Figure 4) showed a significant difference between intervals [*F*_Ankle_(2,30.900) = 8.188, *p*_Ankle_ = 0.001; *F*_Knee_(2,29.369) = 5.601, *p*_Knee_ = 0.009; *F*_Hip_(2,27.106) = 13.173, *p*_Hip_ < 0.001]. Ankle and knee moment showed a significant difference between participants [*F*_Ankle_(12,20.887) = 7.496, *p*_Ankle_ < 0.001; *F*_Knee_(12,13.760) = 6.806, *p*_Knee_ = 0.001; *F*_Hip_(12,19.353) = 0.897, *p*_Hip_ = 0.565]. For none of the joint moments, a difference according to trial was found [*F*_Ankle_(3,27) = 0.089, *p*_Ankle_ = 0.965; *F*_Knee_(3,27) = 2.567, *p*_Knee_ = 0.075; *F*_Hip_(3,27) = 1.447, *p*_Hip_ = 0.251].

An interaction trial × participant was also significant for the ankle moment [*F*(27,54) = 3.875, *p* < 0.001].

*Two tailed pairwise *t*-Test*. To measure whether the steady-state configuration was resulting only from biomechanical factors, we analyzed the intervals after the threshold acceleration time constant (Mid and End). We could not find a difference between body acceleration and velocity between the Mid and End interval (mean ± SEM, vel_Mid_ = 0.002 ± 0.004 m/s, vel_End_ = 0.003 ± 0.004 m/s, *p* = 0.92; acc_Mid_ = −0.001 ± 0.007 m/s^2^, acc_End_ = 0.009 ± 0.005 m/s^2^, *p* = 0.22).

As shown in Figure 3E–G, CoG_AP–Ankle_ and CoG_AP–Hip_ showed a significant difference between the two intervals (CoG_AP–Ankle_Mid_ = 47.27 ± 4.08 mm, CoG_AP–Ankle_End_ = 58.33 ± 5.29 mm, *p*_Ankle_ = 0.0062; CoG_AP–Hip_Mid_ = 1.77 ± 5.23 mm, CoG_AP–Hip_End_ = −5.33 ± 5.21 mm, *p*_Hip_ = 0.0428). CoG_AP–Knee_ did not show a significant difference between the two intervals (CoG_AP–Knee_Mid_ = 25.83 ± 7.61 mm, CoG_AP–Knee_End_ = 32.29 ± 6.89 mm, *p*_Knee_ = 0.0655).

As shown in Figure 4, the joint moments significantly changed between the two intervals (*p*_Ankle_ = 0.0023; *p*_Knee_ = 0.0008; *p*_Hip_ = 0.0001). The ankle moment changed from M_Ankle_Mid_ = 17.10 ± 4.36 Nm to M_Ankle_End_ = 26.33 ± 5.26 Nm. The knee moment changed from M_Knee_Mid_ = 17.62 ± 5.13 Nm to M_Knee_End_ = 33.98 ± 5.82 Nm. The hip moment changed from M_Hip_Mid_ = 12.81 ± 10.50 Nm to M_Hip_End_ = −31.19 ± 5.54 Nm.

## Discussion

In this study, the tight control of lower limb joint configuration was measured in healthy adults when transitioning to standing after walking. Previous work has demonstrated that neural control is required to maintain the location of the line of gravity with respect to the ankle joint (Morasso and Schieppati, 1999; Mirbagheri et al., 2000; Loram and Lakie, 2002; Casadio et al., 2005; Loram et al., 2005, 2007; Kiemel et al., 2008, 2011). In perturbed standing, also the location of the line of gravity with respect to the hip joint is controlled (see hip strategy, Horak and Nashner, 1986). In this study, we measured slow changes in lower limb joints configuration and repeatable steady-state standing configuration within an individual. Although differences in lower limb configuration between initiation of standing and steady-state could be expected to decelerate the body after walking, we measured subsequent changes in configuration that are not mechanically required to maintain standing (between Mid and End intervals). Here, we discuss the possible reasons for the changes in lower limb configuration.

### Misaligned Joint Reference in Quiet Standing

At initiation of standing, we measured fast body deceleration as prompt regulation of acceleration is necessary to remain standing without taking steps after walking. After this deceleration, on average, the lower limb joints became progressively more misaligned with the vertical (Mid vs. End intervals, ankle and hip statistically significant, knee showed a trend). Because we allowed the participants to walk freely in the laboratory before coming to a standing position on the force platform, we can suggest that the observed steady-state misalignment is independent of the body configuration at end of walking. Finally, our analysis could not find a significant difference between the trials performed by the participants. Although a lack of significant difference needs to be cautiously interpreted, the fact that we could not find differences despite intervening experiments and breaks, which are likely to increase variability between trials, may suggest that the data is consistent and that the misalignment is not random.

It is well known that the ankle is misaligned with the vertical in standing, but the result that also the knee and hip configuration became progressively more misaligned was unexpected and requires further explanations.

Because misalignment induces a higher external gravitational moment at the joint, there is no mechanical explanation for the transition in configuration observed here. In feedback control theory it is accepted that movement is controlled via a pre-programmed combination of set points, thresholds, and feedback gains associated with maintaining or changing a configuration of the body. These ideas are common and have been routinely applied to physiological and postural control (c.f. Bernstein, 1967; Fitzpatrick et al., 1996; Maurer and Peterka, 2005; Lockhart and Ting, 2007; Welch and Ting, 2008; Feldman and Levin, 2009). Part of this interpretation is the concept of a set point that the feedback system seeks to maintain or restore following a perturbation. In this experiment, we perturbed human standing by asking participants to walk. We could expect that the body configuration does not change after initiation of standing or, if changes were measured, they were random and not consistent. Instead, we found repeatable changes in configuration, despite different preceding gaits. These changes in configuration suggest that standing was not determined by the end of walking configuration, but other factors were involved in the control of the lower limb configuration. After gait, the body was in a different configuration. The discrepancy between expected and current position could be monitored and minimized (Bays and Wolpert, 2007). We suggest that corrections were made when the relative joint positions were beyond threshold limits, as at initiation of standing. Our hypothesis is that our participants had a body configuration reference which was expected and monitored by the nervous system.

These results do not preclude the established finding that, during long durations of standing, there would be changes in the reference, for example in response to local irritation, fatigue and need for variation (Duarte and Zatsiorsky, 1999; Duarte and Sternad, 2008). However, within the experiment conditions and although the initial joint configuration was perturbed mainly in the direction of the preceding gait phase, we measured adjustments that drove the lower limb joints toward the steady-state standing configuration. We can assume that the body configuration measured at steady-state standing is, therefore, an approximation of the body configuration reference in standing. Although this study’s conclusions are only congruent with the limited number of trials and short duration of standing analyzed, we measured a standing reference which is a misaligned configuration at the lower limb joints.

### Neural Control of Lower Limb Joints in Standing

Investigating how this misaligned standing configuration is maintained is ambitious. Here, we can only draw conclusions and propose speculations based on our data.

We have shown that there is no simple mechanical explanation for the delayed process that we observed between Mid and End intervals. The increased misalignment and joint moments show that steady-state configuration was not necessarily consistent with an energy minimization/optimization principle. This poses a key question: Why participants tend to stand in a more misaligned configuration?

The steady-state misaligned configuration could be consistent with an end of range joint flexion/extension that allows passive stabilization through joint and ligaments locking (close packed). This configuration allows energy conservation because the congruency between articular surfaces allow load distribution and minimizes the energy required to maintain a posture. We could not measure whether the participants maintained a close packed joint position at the end of their flexion/extension range, but our results show that the misaligned configuration could be achieved through modulation in joint moments (Figure 4) which allows small body sway around an average position. This possibility is consistent with proprioceptive mechanisms of standing.

In standing, joint positions have to be sensed, otherwise internal and external perturbations may destabilize the body and lead to loss of balance. It is unclear whether proprioception of small, postural joint rotations is improved by lower modulation of muscular activity (Hulliger et al., 1982; Cody et al., 1986; Di Giulio et al., 2009; Loram et al., 2009) or by slight tonic activity (Fitzpatrick and McCloskey, 1994). However, proprioception is ambiguous when sensing absolute position, rather than its change (Proske and Gandevia, 2012). On the other hand, the nervous system is exceptionally sensitive to central estimation of muscle forces and movement responses to maintain equilibrium (Fitzpatrick and McCloskey, 1994). In this framework, muscle activation involved in modulating joint moments provides an estimate of the mean body configuration. The configuration thresholds and reference could be coded in terms of muscle activation patterns. Our hypothesis is that the muscle activation at a certain point in time could be compared to the reference activation patterns, and muscle activation would be modulated to facilitate standing control. We suggest that this mechanism may be involved in quiet standing.

It is noteworthy that differences could be seen between participants. Investigating these differences and their functional implications is beyond the scope of this study. However, it is possible that particular training techniques, injuries or compensatory mechanisms may be at the basis of this kind of differences, and the reference muscular activation pattern could be different between individuals.

### Limitations

In this study, we did not find a statistical difference between trials and we suggest that this may confirm that the misalignment measured is consistent within a participant. However, the number of trials available per participant varied between 2 and 4 due to technical problems that were only discovered during post-processing. We have interpreted this result cautiously, but the fact that no differences were found despite an intervening long break led us to conclude that consistency and repeatability of the data is acceptable. Furthermore, we chose to treat Trial as a fixed factor. One may consider Trial as a random factor because there is no meaningful, consistent difference between the levels. However, in order to consider Trial as a random factor, Trial needs to be an instance from a large number of repetitions that have been conducted, chosen at random from a larger subset of similar repetitions. This was not the case in the current study because there was an intervening experiment and this is the reason of our statistical model set-up.

Based on the measurements and analyses conducted, we suggest that other factors rather than passive and biomechanical factors are involved in standing. It is difficult to distinguish between active and passive mechanisms at the transition between initiation and steady-state standing, particularly because of the body inertia and the possible non-linear muscle behavior during the transition. This experiment was designed to test whether biomechanical and passive mechanisms could fully explain body position transition between initiation and steady-state standing. In the analysis used here, we aimed to measure changes beyond body deceleration and inertia. This is the reason why we calculated the time constant of body acceleration and we used 3 × SD and we reported changes between the “Mid” and the “End” intervals, rather than the “Beg” interval. Despite this analysis, one limitation is that other non-active mechanisms may be still involved in the transition, but here, we suggest that the increased misalignment and joint moments at steady-state standing cannot exclude an active control of configuration. Further experiments are needed to confirm this suggestion, but in this study we were able to use an ecological protocol and measure physiological mechanisms that are consistent with the hypothesis of active knee and hip control in quiet standing.

## Conclusion

In this study, we measured lower limb joint configuration in standing after walking as a way to physiologically perturb this configuration. We found that the misalignment between line of gravity and ankle, knee and hip and the joint moments were larger at steady-state standing. We, therefore, suggest that the human lower limb joints are controlled relative to a misaligned standing reference. Although the experimental data presents limitations due to trial duration and number of trials, we measured increased joint moments between two intervals after initiation of standing (Mid and End). Because there is no need for a modulation of knee and hip moments in quiet standing, our results suggest that muscle moments were modulated to achieve and maintain the steady-state standing configuration. Additional work is needed to support the current evidence, possibility involving modelling of this tight control in standing. Here, we suggest that modulation of joints moments constitutes an additional voluntary control mechanism, other than the well-established passive and tonic control mechanisms, involved in maintaining quiet standing in humans.

## Ethics Statement

Participants gave written informed consent to these experiments which were approved by the ethics committee of the Institute for Biomedical Research into Human Movement and Health, Manchester Metropolitan University and conformed to the standards of the Declaration of Helsinki.

## Author Contributions

All authors contributed to design of the work and critically reviewed the intellectual content. IDG contributed to acquisition, analysis and drafting of the work. IDG and VB contributed to manuscript writing. The authors approved the final version of the manuscript and agree to be accountable for all aspects of the work in ensuring that questions related to the accuracy or integrity of any part of the work are appropriately investigated and resolved.

## Conflict of Interest Statement

The authors declare that the research was conducted in the absence of any commercial or financial relationships that could be construed as a potential conflict of interest.
